# Assessment of psychosocial functioning and its risk factors in children with pectus excavatum

**DOI:** 10.1186/1477-7525-9-28

**Published:** 2011-05-04

**Authors:** Yi Ji, Wenying Liu, Siyuan Chen, Bing Xu, Yunman Tang, Xuejun Wang, Gang Yang, Liming Cao

**Affiliations:** 1 Department of Pediatric Surgery & Center of Children Medicine, Sichuan Academy of Medical Sciences/Sichuan Provincial People's Hospital, Chengdu, 610072, China; 2 Department of Pediatric Surgery, Children's hospital of Fudan University, Shanghai, 201102, China; 3 Research Institute of Pediatrics, Children's hospital of Fudan University, Shanghai, 201102, China

## Abstract

**Background:**

Psychosocial functioning is poor in patients with pectus excavatum (PE). However, a comprehensive understanding of this issue does not exist. The aim of this study was to assess the severity of psychosocial problems as associated with PE, as well as to identify its risk factors.

**Methods:**

A comparative study was performed at the Sichuan Academy of Medical Sciences/Sichuan Provincial People's Hospital in Chengdu, China. Patients age 6 to 16 who admitted to the outpatient department for the evaluation or treatment for PE were included in the study. In addition to parental reports of child psychosocial problems on the Achenbach Child Behavior Checklist (CBCL), parents also filled in other structured questionnaires, including socio-demographic variables, patients' medical and psychological characteristics. The severity of malformation was assessed by CT scan. For comparison, an age- and gender- matched control group was recruited from the general population. The socio-demographic and scores on CBCL were compared between patients and control subjects. Univariate and multivariate analysis were performed to examine risk factors for psychosocial problems in patients.

**Results:**

No statistically significant differences were found with respect to social-demographic variables between children with PE and control subjects. Compared with control subjects, children with PE displayed higher prevalence of psychosocial problems in the different scales of the CBCL questionnaire such as 'withdraw', 'anxious-depressed', 'social problems' and 'total problems'. Both univariate and multivariate analyses suggested that age, severity of malformation, and being teased about PE were significantly associated with patients' psychosocial problems.

**Conclusions:**

The information derived from this study supports the opinion that children with PE have more psychosocial problems than children from the general population. Multiple medical and psychosocial factors were associated with patients' impairment of psychosocial functioning.

## Background

**Figure 1:**
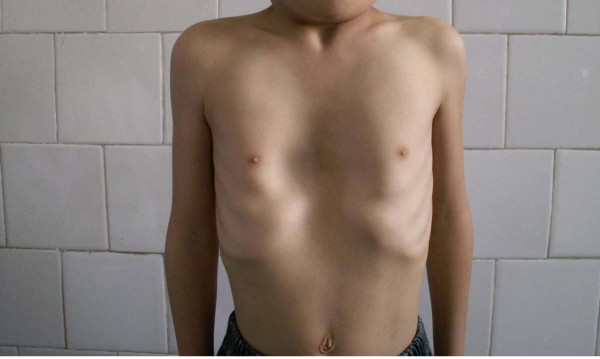
**Chest of a 8-years-old boy with asymptomatic PE**.

Pectus excavatum (PE) is the most common chest wall malformation and one of the most frequent major congenital anomalies. It approximately occurs in up to 1 in 300 to 1 in 1000 births, with a 4:1 male predominance [1,2]. In patients with PE, the sternum and adjacent chest wall are displaced posteriorly toward the spine, creating a depression of the anterior chest wall (Figure 1). Clinically, in many patients with PE, the deformity does not cause significant physiological dysfunction. However, the cosmetic disfigurement can cause a serious loss of self-esteem and affect social behaviour. Precisely when the patient is establishing an independent identity, choosing a trade, or beginning involvement with the opposite sex, he or she is afflicted with a deformity that reduces his or her capacity to do those things.

Yet, despite well documented [3-9] importance of surgery to improve psychological distress and self-esteem in patients with PE, the psychosocial status has not been systematically studied in patients who are not currently undergoing surgical treatment. Additionally, questions exist as to whether clinical features are associated with psychosocial problems in PE patients. To address these issues, the current study examined the emotional and behavioural problems of PE patients by using Chinese Mandarin version of the CBCL, and investigated the risk factors of psychosocial problems by using univariate and multivariate analysis. It was anticipated that children in the control group would report lower frequency of psychosocial difficulties than children with PE. We believe that a better understanding of the features associated with psychosocial problems might improve assessment of emotional risk and assist in the development of intervention and prevention strategies.

## Methods

### Subjects

The study was conducted in Department of Pediatric Surgery & Center of Children Medicine, Sichuan Provincial People's Hospital between June 2007 and August 2010. The patient group contained 415 children between 6 years and 16 years who had PE. They came to our outpatient department, for the evaluation or treatment of PE. Exclusion criteria for subjects included the presence of recurrent PE, pectus carinatum, plat chest, Poland syndrome, or other complex anomaly. For comparison, 400 age- and gender- matched peers were selected from the general population.

This study was approved by the facility ethics committee. Children and parents were informed about the study and written informed consent form was obtained from parents.

### Measurement

The profiles of the patients and their parents were assembled by authors during clinical interviews. Parents were interviewed first, after which they were asked whether they would agree to their children participating in the study. If the parent agreed, the interviewer talked with the child and his/her parent about the study. The child's verbal consent was obtained and audio recorded after it was determined that the child understood what he/she was consenting to. For control group, the questionnaires interviews were conducted at schools or in the participants' own houses, according to the participants' request.

Psychosocial functioning was assessed in detail by Child Behavior Checklist (CBCL) [10]. The CBCL for ages 4 to 18 years (CBCL/4-18) includes competence items and problems items. The problems items can be completed by most parents in about 10 minutes so as to give a description of their children's behavior problems. For each problem item, parents circle 0 if the item is not true, 1 if the item is somewhat or sometimes true, and 2 if the item is very true or often true. The problem items yield scores on three broad band scales and eight narrow band subscales (syndromes). The total problems scale, the externalizing behavior problems scale, and the internalizing behavior scale are broad band dimensions. The narrow band subscales are withdrawn, somatic complaints, anxious/depressed, social problems, thought problems, attention problems, delinquent behavior, and aggressive behavior. A Chinese Mandarin version of the 1991 CBCL/4-18 was used in this study. Previous studies confirmed its acceptable reliability and discriminant validity in Chinese children [11,12]. Here, the total problems scale and all narrow band subscales were used. Children received scores in the clinical range are considered clinical. Reference values are provided by a healthy community sample of 24013 Chinese children and adolescents between the age of 4 and 16 years. The cutting points for clinical designation are based on raw scores. 12.9% of the children had scores in the clinical range for total problems [13].

Parents of all children also filled out a questionnaire on family socio-demographic characteristics, including their gender, age, education, marital status and household income. Information gathered from patients and their parents included severity of malformation and the age which an awareness of the depression arose in the child (patient first perceived his/her deformity). The relationship of appearance-related teasing and poor psychosocial functioning has been documented in the literature: the experience of teasing in particular appears to have a detrimental effect on the young person's psychosocial functioning [14]. Therefore, they also answered questions about whether they had been teased about their chest wall deformity. If the patients and parents gave the same answer, at least that information could be included. All of the interviews were conducted by authors. They had been trained in interviewing and administering questionnaires. Answers were recorded and collected after a consensus conference.

**Figure 2:**
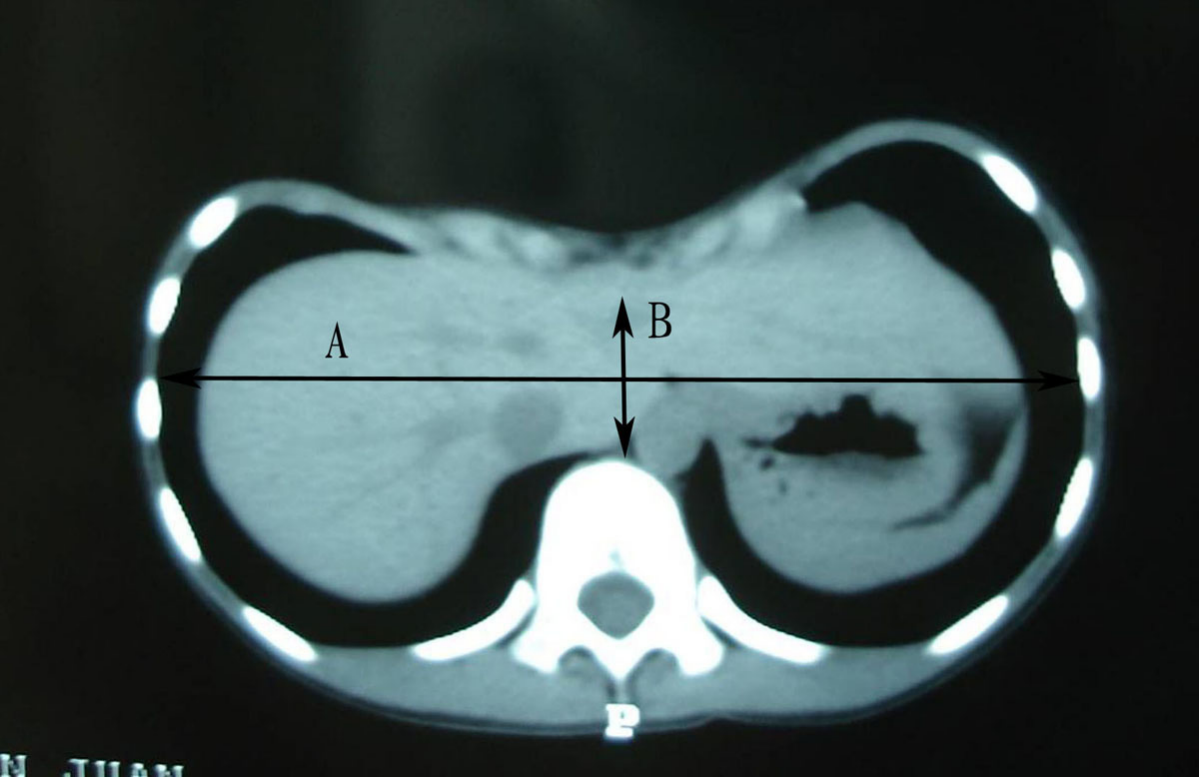
**Haller Index**. The Haller Index (HI) is defined as the ratio of the maximum internal transverse diameter of the chest (A) and the minimum anteroposterior diameter at the same level (B).

The severity of malformation in patients with PE was assessed by Haller Index. It's a severity index, based on measurements obtained from a CT scan of the chest, has been advocated as an objective method of determining the depth of the deformity in PE patients [15]. This index is derived, as shown in Figure 2, by dividing the maximum internal transverse diameter of the thorax (A) by the vertebral-sternal distance at the most depressed position of the deformity (B). It can accurately reflect the true degree of depression in the patients. Several studies have used it to assess the severity of malformation in PE patients [8,16]. Referring to Malek's classification which based on Haller Index [17], the severity of deformity is classed grade 1 to 4.

### Statistical analysis

Statistical analyses were performed using SPSS13.0 (SPSS, Inc, Chicago, Ill) and Microsoft Excel (Microsoft Corp, Pedmond, Wash) in this study. For categorical variables, data were compiled as frequency and percent, and the difference between patient and control groups were compared by χ^2 ^test. For continuous variables, data were calculated as mean **± **standard deviation, and the 2 groups were compared by independent *t *test. In order to clarify the risk factors for the development of psychosocial problems, χ^2 ^test was used to evaluate the correlation between psychosocial problems and independent variables. Significant risk factors were entered into a forward selection multivariate logistic regression analysis. The multivariate logistic regression analysis was performed to calculate the odds ratio and to examine the effect of each factor on the risk for psychosocial problems. A value of P < 0.05 was considered statistically significant.

## Results

### Sample Characteristics

The material is described with frequencies, percent and mean values. Of the 415 PE patients, 38 patients discontinued due to non-compliance, 40 patients were later found to be ineligible and excluded from analysis, leaving 337 eligible patients. Of the control group, 370 children were found to be eligible for further study. Table 1 summarized the demographic variables of the children and parents in 2 groups. No statistically significant were found with respect to age, gender, education, marital status, and household income.

**Table 1:** Demographic Characteristics of Children and Their Parents

Variables	Patient group	Control group	*P*
			
	*n*=337	*n*=370	
Children			
Age*	10.9(2.8)	11.0(2.5)	0.61
Gender†			
Male	266(78.9)	290(78.4)	0.95
Female	71(21.1)	80(21.6)	
Education†‡			
Non-attendance	10(3.0)	6(1.6)	0.68
Primary school	232(68.8)	253(68.4)	
Secondary school	95(28.2)	111(30.0)	
Parents			
Age*	38.1(6.6)	38.5(7.4)	0.45
Gender†			
Male	143(42.4)	148(40.0)	0.51
Female	194 (57.6)	222(60.0)	
Marital status†			
Marries	306(90.8)	332(89.7)	0.78
Divorced	30(8.9)	35(9.5)	
Widowed	1(0.3)	3(0.8)	
Education†‡			
Lower education	96(28.5)	101(27.3)	0.86
Intermediate education	138(40.9)	150(40.5)	
Higher education	103(30.6)	119(32.2)	
Household income per year (RMB) †			
<20000	72(21.4)	69(18.6)	0.67
20000-30000	185(54.9)	193(52.2)	
>30000	80(23.7)	108(29.2)	

*Data given as mean (SD)

†Values are presented as number (percentage)

‡Children's education in the Mainland China is a state-run system of public education. The government provides primary education for six years, starting at age 6 or 7, followed by six years of secondary education for ages 12 to 18. Some of the children aged 6 did not attend school.

Parents' educational level is divided into lower, intermediate and higher education. Lower education then involves elementary education, general secondary education junior-level, lower vocational education; Intermediate education involves general secondary education-senior level, and vocational education-junior level; Higher education involves vocational education-senior level and university education.

The majority patients had no obvious physiological dysfunction in daily life. 21 (6.2%) patients reported shortness of breath, chest pain, and other similar symptoms that affect their activity level or occur with mild exertion and limited exercise performance. Computed tomography (CT) scans were performed in all patients to document the severity of the deformity. 50 patients had grade 1 deformity with a HI of 3.0 to 3.9, 149 patients had grade 2 deformity with a HI of 4.0 to 4.9, 68 patients had grade 3 deformity with a HI of 5.0 to 5.9, and 70 patients had grade 4 deformity with a HI ≥ 6.0. Finally, 56 patients refused to have surgical repair for a lack of physical symptom or a fear of surgery. The HI in patients who did not want surgery (4.3 ± 1.2) was significant lower than that in those who did (4.8 ± 1.1). (*P *< 0.01)

### Psychosocial problems in relation to PE

**Figure 3:**
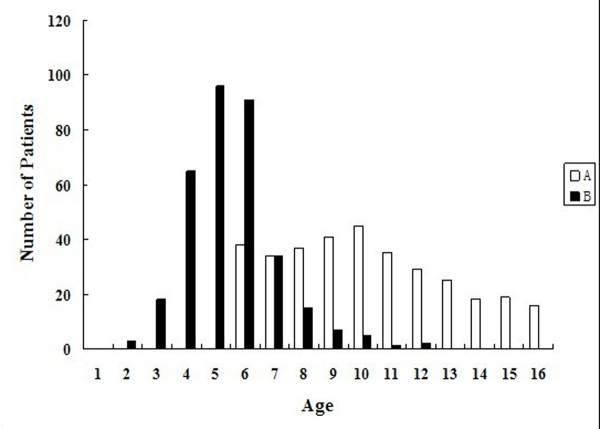
**Frequency distribution of patients' age**. Of all patients, 252 patients first perceived their deformity when they were 4, 5 or 6 years old. Only 3 patients first perceived the chest wall deformity older than 10 years. A: Patients' age at time of the study. B: Age at which patient became aware of pectus excavatum.

252 (74.8%) patients first perceived their deformity when they were 4, 5 or 6 years old. Only a few patients recalled that their chest depression was not obvious when they were young, and they didn't notice their deformity before the age of 10 (Figure 3). According to parental and patient recall, 198 patients (58.8%) found their deformation by themselves, 139 (41.2%) patients were told about their chest deformation by others, mainly their parents. Dissatisfaction and being teased about their deformity were the motivation factors for treatment among patients: 125 (37.1%) patients admitted that they had asked their parents to take them to hospital at least once in the past year, most of them are older than 10. 147 (43.6%) patients said they had tried hard to avoid exposing their chest in public places. 77 (22.8%) patients reported that they had been teased about their chest deformity, "often or sometimes", with 97.4% teasing done by peers and 2.6% by adults outside the family. No one had experienced this kind of behavior from family members or extended family.

We calculated the scores of related items with CBCL scales for each child. Table 2 presents the prevalence of psychosocial problems of patient group and control group. At the subscale level, from 4.45% to 8.01% of patients generally received scores in the clinical range in each subscale. The prevalence of withdrawn, anxious-depressed, social problems was significantly higher among patients than control subjects. For total problems scale, the corresponding data of patients group and control group were 19.58% and 12.70% respectively. No difference was found with respect to prevalence of total problems between children in the general population and control subjects (*P *> 0.05). Patients group were significantly more likely than the control group to demonstrate total problems (*P *< 0.05). There were no statistically significant differences in other scales between two groups.

**Table 2:** Prevalence of psychosocial problems in patient group and control group

CBCL scale	Patient group	Control group	χ^2^	*P*
				
	*n *= 337	*n *= 370		
Withdrawn	23(6.82)	12(3.24)	4.808	0.028*
Somatic complaints	15(4.45)	8(2.16)	2.692	0.087
Anxious/depressed	27(8.01)	15(4.05)	4.944	0.026*
Social problems	21(6.23)	9(2.43)	6.264	0.012*
Thought problems	17(5.04)	12(3.24)	1.455	0.228
Attention problems	16(4.75)	13(3.51)	0.683	0.409
Delinquent behavior	19(5. 64)	11(2.97)	3.038	0.079
Aggressive behavior	21(6.23)	13(3.51)	2.846	0.092
Total problem	66(19.58)	47(12.70)	6.220	0.013*

Data are presented as number (percentage).

*The differences are statistical significant if *P *< 0.05.

### Factors associated with psychosocial problems

Table 3 summarise the univariate analyses examining relationships between patients' characteristics (gender, age, severity of malformation, first perceived age, perceived approach, being teased, whether had surgical repair or not, parents' education level, and household income) and prevalence of the psychosocial problems (total problems). These analyses highlight the apparently significant impact of patient characteristics on poor psychosocial functioning: compared with the age group under 9 years, the age group12-16 was at higher risk for psychosocial problems; the patients with severe deformity (HI ≥ 6.0) were significantly more likely than the patients with mild deformity (3.0 ≤ HI ≤ 3.9) to have psychosocial problems; patients with lower maternal education were significantly more likely than patients with higher maternal education to have psychological symptoms; the frequency of psychosocial problems was significant greater in patients who have been teased by others. Based on the statistically significant difference uncovered in univariate analysis, the results of multivariate regression analysis indicated that age of patients (OR 1.565, CI 1.080-2.269), severity of malformation (OR 1.414, CI 1.067-1.875), and being teased (OR 2.941, CI 1.528-5.659) were associated with psychosocial problems. Mother's education (OR 0.015, CI 0.949-2.038) was not a significant predictor of psychosocial problems of PE patients (Table 4), even if it exhibited a significant association on univariate analysis.

**Table 3:** Risk factors for the psychosocial disorder derived from univariate analysis

Variable	n	Total problems		χ^2^	P	Odds ratio	95%Confidence Interval
						
		**No**.	(%)				
Age							
6-8 years	109	16	14.7				
9-11 years	121	21	17.4	0.304	0.581	1.221	0.601-2.480
12-16 years	107	29	27.1	5.053	0.025*	2.161	1.094-4.268
Gender							
Male	266	49	18.4	1.085	0.298	1.394	0.745-2.610
Female	71	17	23.9				
Severity							
I Degree	50	7	14.0				
II Degree	149	24	16.1	0.126	0.722	1.179	0.475-2.931
III Degree	68	14	20.6	0.855	0.355	1.539	0.591-4.294
IV Degree	70	21	30.0	4.174	0.041*	2.633	1.020-6.796
Perceived age†							
≤ 6	271	50	18.5	1.131	0.288	0.707	0.372-1.342
≥ 7	66	16	24.2				
Perceived approach							
By themselves	186	39	21.0	0.504	0.478	1.218	0.706-2.103
By others	151	27	17.9				
Being teased							
Yes	77	22	28.6	5.118	0.024*	1.964	1.087-3.547
No	260	44	16.9				
Had surgical repair							
Yes	281	57	20.3				
No	56	9	16.1	0.526	0.468	0.573	0.348-1.625
Father's education							
Higher	115	19	16.5				
Intermediate	134	26	19.4	0.347	0.556	1.216	0.634-2.335
Lower	86	21	24.4	1.925	0.165	1.632	0.814-3.274
Mother's education							
Higher	92	14	15.2				
Intermediate	143	25	17.5	0.241	0.624	1.196	0.586-2.440
Lower	100	27	27.0	3.961	0.047*	2.061	1.003-4.234
Household income per year (RMB)							
>30000	80	14	17.5				
20000-30000	185	34	18.4	0.029	0.865	1.061	0.534-2.109
<20000	72	18	25.0	1.283	0.257	1.571	0.716-3.447

Note: Some parents did not answer all questions, resulting in different totals.

*The differences are statistical significant if *P *< 0.05.

† The age which an awareness of the depression arose in the child.

**Table 4:** Multivariate regression analysis to identify risk factors associated with psychosocial disorder

Variables	OR	CI (95%)	β	S.E. (β)	Wald χ^2^	P
Age	1.565	1.080-2.269	0.448	0.189	5.598	0.018*
Severity	1.414	1.067-1.875	0.346	0.144	5.803	0.016*
Being teased	2.941	1.528-5.659	1.079	0.195	10.435	0.001*
Mother's education	0.015	0.949-2.038	0.330	0.195	2.864	0.091

*The differences are statistical significant if *P *< 0.05.

## Discussion

We studied the psychosocial functioning of 337 children aged 6 to 16 years old who were born in China. The subjects included not only the children who would choose the surgery but also the children who would not. Our findings demonstrated that 74.8% of children with PE discovered they were different around the age of 4 to 6. This is when name-calling and teasing begins, and the children learn what it means to be different. Their first problem tended to be not an organic but a psychosocial one, caused by self-awareness of the deformity and/or by being teased by others. Many children, especially some older children want the deformity corrected as soon as possible.

As identified by Lavigne et al [18], there is wide variation in the psychosocial functioning of children with a chronic illness. In this study, assessment of psychosocial functioning was achieved by CBCL. We found that children with PE had significantly more emotional and social problems as reported by their parents on standardized question, in comparison with children in the general population. This result is comparable with previous reports of PE-induced deterioration of psychosocial functioning across a wide age range [6,19]. Meanwhile, we found that children with PE did not have more behavioural problems than control group. This finding is also in line with studies of psychosocial functioning in children with PE [8,19], but contradictory to studies on children with congenital microtia or hypospadias [20,21]. We presume that this may be attributed to the different diseases have different risk factors, which have different relative impacts on psychosocial functioning [18].

However, one limitation of the study was that the study sample was entirely composed of patients seeking medical evaluation or treatment for PE and cannot be considered representative of the general population of PE subjects. In this respect, poor psychosocial functioning could be a motivation for referral and higher scores are usually observed in clinical-based samples when compared with population-based surveys. Another limitation of the study is that there had no reference values for externalizing problems and internalizing problems in our community [12,13]. For this reason, we can not present the prevalence of psychosocial problems which are based on these two scales. Although the CBCL can be complemented by the Youth Self-Report form (YSR), the YSR is only constructed for children aged 11 years and older, and thus would not be suitable for use in the current study focused on children from age 6. Furthermore, the information on child emotional and behaviroal problems was obtained by questionnaires, and not by semi-structured clinical interviews conducted by clinical professionals. Although the patients who had higher scores on CBCL scales were recommended for further assessment in psychiatric outpatient department, identification of children in control group is typically impossible due to ethical reasons for protecting anonymity.

On the other hand, this study provided one of very few representative samples of PE patients. Information from a standardized behavioural checklist provided a wealth of information about the psychosocial status of children with PE. Moreover, we compared the patients with normal children matched for sex and age, and all subjects resided in the same health care catchment areas and assessment were carried out in closely related time periods in both groups. Therefore, the study of these children could provide a more accurate picture of PE individuals referring to clinical practices, and provide relevant clues for treatment programs. We, like others, believe that poor body image and impaired psychosocial functioning should be an enormously important concern for surgical repaired and/or psychosocial intervention [8,22,23].

This study showed a tendency for the prevalence of psychosocial difficulties to increase with age in patients who have not had surgery. It would seem to reflect the increasing burden of psychosocial trials which individuals face as they grow older. In other words, if PE patients do not have psychotherapeutic help or surgical repair, if they are left alone with their difficulties, psychosocial problems may increase with age. From the psychological point of view, the early surgery is ideal. Additionally, mounting evidence has emerged to suggest that many patients who do not undergo repair of severe PE deformities in childhood will experience worsening symptom in their adult lives [24,25]. However, there is debate in the literature regarding the best age for repair. Some authors argued that repair at too early of an age may result in improper growth of the chest wall and other complications, so the surgery should be performed during the stage of teenage (close to the age of skeletal maturity) when the operation for correction of PE can be readily accomplished [26]. In contrast, a vast amount of research has shown that continuous technical refinements have significantly decreased the complication rates and postoperative morbidity, and Nuss procedure has been demonstrated to be effective for quality repair of PE patients aged from 3 to 50 years [7,27-31].

Within the study of visible or invisible (i.e.: normally hidden by clothing) difference or deformity, the presumption that more significant pathology is associated with greater psychological distress is strongly held. It is reasonable to expect that a person with extensive scarring, for example, may experience greater difficulty than a similar person with fewer scars [32,33]. In our study, univariate analysis showed that the severity of PE was significant associated with psychosocial adjustment. Multivariate logistic regression analysis also identified it as a risk factor for psychosocial adjustment. This is a fascinating finding, providing very strong support for the inclusion of objective measures of appearance in studies exploring the psychosocial impact of PE, particularly where this includes an evaluation of treatment. The result is consistent with finding from Ohno et al.'s study [16] which included a group of PE patients who have not undergone the surgery. However, this finding stands into contrast with another finding that there was no significant relationship between the severity of PE and mental health [8]. We presumed that contradictory results may be attributed to the following reasons: first, in previous study, patients who did not undergo surgery were excluded from the psychosocial study. In fact, patients with mild/moderate deformations are associated with small loss of function and little focus on the appearance related aspects of the PE, their parents may be reluctant to take the risk of surgery unless the symptoms seem to be getting worse. This may lead to the reduction in psychosocial morbidity of the patients with mild/moderate PE in clinical research. Second, the psychometric testing tools in previous study were distinctly different from ours: in previous study, parents were interviewed by the Pectus Excavatum Evaluation Questionnaire (PEEQ). However, the PEEQ was developed not for the detection of psychosocial problems but mainly for the assessment of impact of surgical repair [4,5].

Teasing is seen as a negative and repetitive action committed by one or more person and is associated with a host of negative consequences for children. The results of current study indicate that approximately 22.8% of patients had been teased about their PE by others, mainly their peers. We find that children with PE report having been teased less frequently than children with facial anomalies [34]. This may due to the fact that the upper part of the body is expected to be covered with clothes. Furthermore, many children with PE tried to avoid exposing their chest in public places because of the fear of being teased, and 'an unwillingness to be seen without a shirt while swimming and participating in sport or social activities' is the most frequently quoted complaint [22]. Unfortunately, researchers have found that the experience of being teased and the fear of being teased can result in anxiety, emotional distress, and difficulties with social integration [14,35,36]. In this study, teasing was associated with significantly higher prevalence of psychosocial difficulties in patients. This finding is not surprising given that our results indicate teasing history is greater for PE children with psychosocial problems. Surgeons and pediatricians need to be aware that psychosocial functioning of children with PE might be impaired by teasing remarks from peers.

## Conclusions

In summary, this study examines a relatively large sample of patients with mild to very severe PE. The result shows that children with PE experience significantly decreased psychosocial functioning in emotional and social domains of the CBCL. Moreover, we find that age, severity of malformation, and being teased about PE has significant effects on psychosocial problems. Thus, a better understanding of the influence of PE on psychosocial functioning, including the identification of patients most vulnerable to psychosocial impairment, is acquired. This improved understanding could enable the application of strategies in both clinical practice and public health that seek to prevent further psychosocial problems associated with PE patients. Clinicians involved in care for patients with PE should be aware of the potential factors associated with psychosocial functioning. Studies looking at psychological intervention to ameliorate psychosocial problems in these patients are also warranted.

## Competing interests

The authors declare that they have no competing interests.

## Authors' contributions

YJ contributed to the conception and design of the study, the conception and interpretation of the statistical analysis, and drafted the manuscript. YWL contributed to the study design, drafted the paper and revised of the manuscript. YSC conducted the statistical analysis, contributed to the interpretation of data, the drafting and revision of the manuscript. BX, MYT, JXW, GY, MLC contributed the acquisition of data, the interpretation of the statistic analysis, and revised the manuscript. All authors read and approved the final manuscript.
